# Electrolysis in Morbid Alterations That Are Produced in the Prostate by Gonorrhoea of the Urethra

**Published:** 1889-01

**Authors:** John D. S. Davis

**Affiliations:** Birmingham, Ala.


### ELECTROLYSIS IN MORBID ALTERATIONS THAT ARE PRODUCED IN THE PROSTATE BY GONORRHŒA OF THE URETHRA.*

BY JOHN D. S. DAVIS, M. D., OF BIRMINGHAM, ALA.

The structure of the prostate gland consists in greater part of muscular substance, tubular glands and glandular tissue.

Like the follicles of the fossa navicularis, the excretory ducts of the prostate gland are ever liable to become diseased through

*Read before the Southern Surgical and Gynecological Association, December 6, 1888.

the propagation of a gonorrhœal discharge to the deeper parts of the urethra, giving rise to a sero-muco-purulent catarrh.

The prostate often is enlarged and often suppurates, the pus tunneling its way into the rectum, urethra or perineum.

The muscular structure of the prostate suffers no pathological alteration; it is in a constant state of contraction, expelling the secretion, and causing tenesmus of the neck of the bladder and anus.

Owing to the capillary engorgement which takes place in the prostate and urethra, the patient is troubled with dripping of urine at the end of each act of micturition.

Patients suffering with gonorrhœal serous or mucous catarrh of the prostate, are generally troubled on the one hand with difficulty in defecation, and, on the other, from a constant desire to urinate.

So long as the œdematous swelling of the prostate is not very intense, the pus corpuscles, originating in the *cryptæ*, become mixed with the normal secretion of the gland, and form with it a gluey, yellowish-green fluid to be thrown off through the urethra.

Electricity, properly applied, will restore the organ to health, and relieve it of a disease that never goes away of itself.

By mild, negative galvanic currents, we have a practical, painless, harmless and convenient means of treating this form of disease.

In cases where the œdematous swelling of the prostate is very tense, even to the complete closing of the urinary track, electricity will often offer the best and simplest route to complete recovery. In cases where there is progressive suppuration, with the cavities of the gland filled with pus and much dilated, galvanic stimulation with varying dosage and polar action, even in such cases of extensive suppuration, will prevent the glandular spaces coalescing; correcting to a very large extent the perversion of nutrition; whether it be a question of hypertrophy or suppuration, the current will be, in experienced hands, a stimulus of the first order, which will tend to re-establish the equilibrium or normal function of the gland.

In cases where there is progressive suppuration with effusion of pus into surrounding tissue, I always make a free incision with the knife through the perinium into the prostate gland to secure drainage. With a staff in the urethra for a guide, I proceed as in an operation of external urethrotomy, extending the incision to and through the prostate gland. This class of cases which, primarily originates from the propagation of the acute purulent urethral gonorrhœa to the prostate gland, and a condition often aggravated from injuries of the prostate by the introduction of catheters, bougies and impaction of calculi, already suppurated, will, after a thorough evacuation of the pus and washing out the cavity, under galvanic stimulation, rapidly return to its natural size and regain its normal function.

Having long followed the teaching of Newman in the treatment of urethral stricture by electricity with good results, I began this method for prostatic troubles, and prepared myself for the investigation of a treatment, the efficacy of which has been much doubted. The following three cases of thirty-seven treated at my office, will suffice to illustrate the efficacy of the treatment herein advocated. In every case care was taken to observe every successful point ascribable to the treatment.

*Case 1.*—Y. C.; locomotive engineer by occupation; twenty-seven years of age, good general health and temperate. Had gonorrhœa six months previous to the time (July 10, 1887), the patient presented himself to me for treatment of enlarged prostate.

He had a constant desire to urinate; difficulty in defection and considerable burning pain in the urethra. The urethra was smooth, and admitted a number 32 French *bougie à boule* to the prostate where it was obstructed. I succeeded in passing a *bougie à boule* number 3, French, through the prostatic urethra. The anterior wall of the rectum was bulged out by a painful tumor, urine alkaline, specific gravity 1025, containing pus.

Newman's insulated olivary bougie, number 3, French, passed into the prostatic urethra. Current five milliampéres passed through bougie, connected by cathode for fifteen minutes. The patient was sent to bed for three days.

July 13.—Slight pain after urinating. Number 10 *bougie à boule*

was passed and engaged in prostatic urethra. The negative current, five milliampéres, was again passed for fifteen minutes. No pain was produced, and no unpleasant symptom followed either application.

*July 16.*—The prostate gland was much smaller, the stricture allowing the easy passage of a bougie number 21, French. In like manner the negative current was used for fifteen minutes. The patient was so much improved that he left and did not appear for treatment again. He took his engine out on the road July 18th. He experienced no difficulty in urinating after the 13th of July.

The gleety discharge ceased on July 15th, and has not since re-appeared. Last examination, September 30, 1880, showed prostate gland almost normal in size, and prostatic urethra admitted a bougie number 32, French.

*Case 2.*—June 1, 1888. W. B.——, tobacco merchant, aged twenty-four years, white, good health and temperate. Had four attacks of gonorrhœa and been under the care of several surgeons for stricture and gleet. Had internal urethrotomy performed three times. The urethrometer showed numerous contractions from the fossa navicularis to depth of four and one-half inches. A *bougie à boule* number 7, French, could be passed to the prostate where it was arrested by a large tumor. The prostatic urethra would not admit the smallest instrument. The membranous urethra unusually granular, bleeding quite profusely on the passage of instruments—could not pass urine.

Aspirated the bladder, drawing off one pint and one-half of alkaline urine; specific gravity 1027; containing pus with phosphates.

A number 7 bulbous bougie was passed to prostate, connected with the cathode, the anode placed over the abdomen; current five milliampéres, was used for five minutes when the current was increased to ten milliampéres and continued for ten minutes, when the bougie slipped through into the bladder. Patient was allowed to remain up and continue his business. Considerable scalding in micturition was experienced for two days.

June 3.—Number 14 bulbous bougie was successfully passed.

June 6, 9, 12 and 14.—Numbers 16, 18, 30 and 32, were successfully passed. The gleety discharge subsided June 13, and had not returned.

Patient examined July 15, the urine slightly acid, specific gravity 1021, no pus. Passed a steel sound number 32, French, readily into the bladder, urine passed in large stream and without difficulty. Last examination November 15, 1888.

*Case No. 3.*—July 9, 1888. D. F——, drummer by profession, aged forty years, white, intemperate. He gave history of repeated attacks of gonorrhœa. He had just had a chill, and was in great agony; difficulty in defecation and inability to urinate. Very nervous, temperature 103, pulse 130 beats per minute. For six hours he had been frequenting the water-closet, straining every muscle and nerve to forcibly expel the urine and defecate. In his repeated acts of muscular contraction by inspiration, endeavoring to compress the bladder by the action of the diaphragm and the pressure of the abdominal wall, the prostate was elevated by the levator ani muscle, and compressed against the symphasis pubis, thus causing still greater compression of the urethra which was already narrowed and entirely prevented the flow of the urine.

The patient was placed on the back and a *bougie à boule*, number 5, French, was engaged in the prostatic stricture, connected with the cathode—current five milliampères for seven minutes—when the urine shot out by the instrument, and the bougie passed rapidly into the bladder.

The urine was allowed to pass, and then the operation was continued for eight minutes longer.

The operation was repeated every third day for four times, when the patient was ordered out on a trip.

July 12.—Fever subsided immediately after first operation, and prostate gland began to diminish in size.

August 21.—The patient was examined, and the prostatic enlargment had almost subsided; no discharge, no pain, and a good, large, round stream of urine passed without difficulty.

November 17th, 1888.—The patient reported himself entirely well.

Thirty-four other cases (embracing six cases of fourteen urethral strictures,) have been consecutively subjected to this method of treatment.

In no instance has the pain been of sufficient severity to necessitate the subsequent use of anodynes.

The utmost delicacy in the manipulation has invariably been exercised, in order that the pain might be eliminated as far as possible.

The length of each seance, fifteen minutes.

The intervals between the sittings, usually three days.

Differing from the action of stricture of the urethra anterior to the membranous urethra, the gland begins to decrease in size, and the calibre of the prostate urethra increases from the first application.

The œdematous swelling that follows two or three days after the use of electricity in stricture anterior to the membranous urethra, often causing passive obstructions, does not take place in the class of prostatic troubles reported in this paper. In urethral stricture the cicatricial tissue does not immediately begin to disappear, but the prostate gland (which is certainly more susceptible to the influence of low currents) begins at once to decrease and continues to complete recovery. The spasmodic action is allayed at once, softening taking place, and the absorptive action begins immediately.

The results which have attended the application of electricity in my thirty-seven cases treated this year, gave me convincing proof of its merits. Supported by a very large experience in the use of electrolysis in strictures of every part of the urethral canal, I must reject any idea that electrolysis does not benefit spasmodic strictures of the urethra. Indeed, it is a fact, that the prostate receives the electric stimulation more kindly, and is benefited more effectually by the contemporary effect of the current; for in other parts of the urethral canal the posthumous effect of the current does the most good with strictures due to enlargement of the prostate, a contemporary and permanent benefit, while in strictures anterior to the membranous urethra an œdematous swelling follows the electrolysis, and often in two or three days produces a complete occlusion of the calibre of the urethra.

Owing to this fact electrolysis can be repeated every two or three days in prostatic troubles, while in urethral strictures elsewhere the surgeon must wait for the disappearance of the œdematous swelling due to the electrolytic effect.

Certainly it assures the patient a rapid and complete recovery. And how, I am unable to conclude, save theoretically.

Heat can only come into existence at the expense of some other energy. The chemical processes that proceed within the quiescent muscle to produce heat are augmented and increased by stimulation. The constituent albuminous bodies composing the muscular prostatic tissue, which cannot be separated by the ordinary method used in chemistry, unite during galvanic stimulation, setting free acids and alkalies (which act according to polarity on the living structure) to produce heat, softening of tissue, decomposition, relaxation and absorption.

The non-nitrogenous bodies, glycogen, fat, salts, acids and myosin unite during active stimulation by faradization, forming lactic and carbonic acids, and alkalies, a strong factor in the rapid degeneration and absorption of abnormal products.

The number of chemical modifications recurring at each electro-tissue-stimulation is always the same, but when a greater stimulant is applied, a larger amount of substances are consumed, so that both the production of warmth and decomposition may, through the stimulant, remain the same, according to the degree of tension and stress of the living tissue. So long as the tissues are uninjured, the matter thus decomposed is carried away and fresh, nutritive, re-productive matter is brought to replace the decomposed or expended material. The more abundantly the blood current flows through, the more rapidly and quickly are the products of decomposition removed and the earlier replaced. It is, therefore, very important to apply the negative (moist) pole. For in the application of galvanic electricity to a living body, a perceptible dilation of the capillaries will result from the application of the cathode, and contraction on application of the anode. Catelectrolysis produces dilation of the capillaries and relaxation of muscular tissue while anelectrolysis produces contraction of muscular tissue.

If a muscle is continuously stimulated, it will be found that the contraction, though at first very considerable, soon decreases and finally entirely ceases to contract altogether. Muscular fibre when thus exhausted by the stimulating effects of the low galvanic current, may be induced to contract after rest, but it requires much time to recover from the exhaustion.

If the muscular tissue has been stimulated until disorganization takes place in the muscular fibres, it may be capable of again becoming active after a long interval of rest, but not until the products added exceed that which was expended. The change which occurs in the muscular tissue of the prostate from electrolysis deprives it of the capacity for contraction for a long time. The same thing happens if blood-current ceases to pass through the tissues, or if prolonged stimulation induces too great a softening and decomposition of its albuminous bodies.

Apart from the enlarged condition of the prostate, there is always an accompanying spasmodic contraction of the muscular tissue of the gland (sphincter vesicæ externus) and a number of tubular glands, in addition to the glandular structure which continues in a state of tetanus, relieved only by an exhausting stimulant. Hence the efficacy of the contemporary effect of electrolysis.

To perform well any operation with galvanism, a good galvanometer is of paramount importance. The absence of a good galvanometor submits the physician to the "caprice of hazard," and "places him in the dilemma of making the operation too weak or too strong, without the means to control it."

Next to a good battery (which should have small cells), armed with a rheostat and reliable galvanometer, the intra-urethral electrode is of greatest importance. The nickel-plated olive-shaped bulbs on slender, hard rubber rods of the proper length and curve, introduced by Newman, have no superior.

The cutaneous electrode should be applied to the abdomen or to any other part of the body that is not very susceptible to the current.

Never oil the urethral bougie or coat it with a non-conducting material, before introduing it into the urethra, as we do other instruments to facilitate their passage along the urethral canal. An electrode, so oiled, would be insulated, and the current not able to pass properly into the structure of the prostate gland. Glycerine being an available conductor, should always be used as a substitute for the common lubricants. In the absence of a good rhoestat, the size of the cell of the battery used is of very great importance, for if they are too large the electrolytic action of the current will be so intensified as to become cauterizing, and thus harm may come to the patient's prostatic urethra. Cells holding one to two ounces of the fluid are large enough. According to the size of cells and elements (zinc and carbon), the action of electrolysis can be regulated so as to produce simply a softening of tissue, decomposition and absorption.

After the introduction of the urethral sound, above all things, examine all the couples of the battery, to see that they are in correct working order, to avoid interruption of the current during the course of the operation.

Close the current on itself and make (in the absence of a good rheostat), each of the couples enter, successively, cell by cell, one by one; the deviation of the compass will serve as a galvanoscope, and reveal, at all times, the integrity of the battery.

There is always a slight shock, even in giving the current cell by cell, to nervous persons, but, in the absence of a rheostat, by increasing cell by cell the shock is reduced to a minimum, where on the contrary, if administered two by two, three by three, the shock becomes too great by the transition to the discomfort of the patient.

In order to obtain the maximum therapeutic effect of this treatment, the current must be minutely and carefully administered.

The cathode must be introduced into the prostatic urethra, and a very weak current passed through it, increasing little by little until the galvanometer registers the required number of milliampéres.

The method of electrolysis is simple and can be executed by the surgeon alone, and without aid. It is painless and requires no anæsthetic. No inconvenience is felt after the operation, and anodynes are never required; it is absolutely harmless; it is antiseptic on account of the energy of the (even very low) current employed, and the electrolysis (called galvano-chemical absorption by Newman) is always followed by a process of retrogression, disintegration and absorption.

A contemporary focus of derivation is created in the diseased gland, analagous to what is done in a profound shock of the muscular system from over-exertion, or otherwise, which continues after the cessation of the current, transforming into posthumous effect, a slow and continuous action, the temporary shock which the passage of the current has transmitted to the muscular element of the prostate gland, and it finally inaugurates a process of retro-action, disintegration and absorption.

The therapeutic indications are identical with those of stricture of the urethra. The time of seance may be double the time required in strictures anterior to the prostatic urethra. So long as the diseased gland is characterized by congestion and hypertrophy gorged with secretions, and allows a considerable quantity of sero-muco-purulent fluid to escape by the urethra, electrolysis will restore it to its normal condition.

The muscular tissues are relaxed by the stimulating effect of the negative current; the vessels are relaxed, and the blood-flow is increased by diminishing the capillary resistance, and absorption readily takes place.

In the treatment of these cases I am often, for want of time, confronted with the proposition to increase the intensity and diminish the seance, to render the same sum of electric outflow, and consequently equalize the therapeutic effects.

Physically speaking, a current of 25 milliampères, during five minutes, is equal to a current of five milliampéres during twenty-five minutes, but the physiological and therapeutical action of these two amounts cannot be compared on account of the varying energy of each current.

In the former a chemico-galvano-cauterization would result and in the latter galvano-chemical absorption. It is my idea that these two terms should, for fear of confusion, be defined as follows:

Galvano-chemical absorption (electrolysis) is a chemical decomposition, which borrows its immediate effects from the suppurated albuminous bodies, the chief constituents of tissue, which generate warmth, relaxation, softening and absorption.

Chemical galvano-cauterization is an absolute chemical cauterization, which borrows its immediate effects from the acids and alkalies, which are the products of electrolysis, acts essentially different in the fact, the current of high intensity which traverses the body, while in the same physiological manner the body furnishes the necessary elements for its own cauterization; the current extracts the acids and bases, which were latent, and held in combination (the non-nitrogenous bodies, glycogen fat, salts, lactic acid, creatin and myosin), and brings them forth with physical and therapeutic consequence of a cauterization that is thermic in appearance and result.

Electrolysis is inseparable from the application of the continuous current to living tissues, and should never have been substituted by galvano-chemical absorption. Absorption, as defined by Webster, is "the process or act of being made passively to disappear in some other substance through molecular or other invisible means, as the absorption of light, heat and electricity."

This definition of absorption is used by Dr. Newman as a correct interpretation of electrolysis. Electrolysis means more, in the fact that its contemporary effect is to soften, decompose and separate the albuminous bodies of the abnormal tissue, in preparation for the posthumous effect, which is that of absorption.

As absorption does not mean all the functions of alimentation (mastication, deglutition, digestion, etc.), when applied to the power of nourishing, so it does not mean all the functions of electrolysis (heat, softening disorganization, retrogression, etc.), when applied to the *materia medica* of this new treatment of strictures, prostatic hypertrophy, etc., by the galvanic current of low intensity.

"Electrolysis," says Apostoli, "is inseparable from every application of the continuous current to the human body, which contains substances especially capable of being decomposed and called electrolysis in the language of physics. It consists in the decomposition of matter, salt, etc.; it is the every-day experiment in the course of lectures on physics, of decomposing water in a voltametre, and of making oxygen and hydrogen gas separate each into a different tube.

"But as the decomposition of salt sets free acids on one side, and bases on the other (acids at the positive pole and bases at the negative), besides this first action, inseparable from the employment of the continued currents, and rightly called electrolysis, we have a second action which will be due to the setting free of the acids and bases, and to the respective cauterization which they produce upon the tissues in presence of which they may be, we shall have, in fact, a chemical galvano-cauterization, either positive or negative.

"The effect of electrolysis is therefore entirely analytical, and prepares for the subsequent caustic action, which is rather synthetical."

Electrolysis expresses the decomposing effect of electricity, which may exist, in a current of low intensity, without the final effect which results from a current of high intensity.

While electrolysis does not in any way prejudice the final effect produced by a galvanic current of high intensity, (chemical galvano-cauterization, either positive or negative,) the primary action of which it always is, it does prejudice the final or second action in a current of low intensity.

The galvanic current of low intensity will produce a decomposition of the tissues of the body, without resulting in or producing chemical galvano- cauterization of the superficial layers.

Electrolysis, while applicable to all strictures of the urethra, is of permanent benefit in many of the morbid alterations of the prostate.

It has an anesthetizing influence upon the terminal nerves of the point of application; it aids in overcoming spasmodic stricture of the prostatic urethra, in early relaxation of spasm by muscular exhaustion, following the continued over-stimulation; it seals the distention and relaxation by natural reproductive processes; it excites absorption and relieves the patient.

